# Supplementary material for: A fault-intrusion-tolerant system and deadline-aware algorithm for scheduling scientific workflow in the cloud
Source: PeerJ Comput Sci. 2021 Nov 2;7:e747. doi: 10.7717/peerj-cs.747 (PMC8576554; doi:10.7717/peerj-cs.747)
Supplement: Supplemental Information 6 [file peerj-cs-07-747-s006.zip › Myproject1.1/docs/org/cloudbus/cloudsim/class-use/DatacenterBroker.html]

Uses of Class org.cloudbus.cloudsim.DatacenterBroker


JavaScript is disabled on your browser.


- Overview
- Package
- Class
- Use
- Tree
- Deprecated
- Index
- Help

- Prev
- Next

- Frames
- No Frames

- All Classes

## Uses of Class org.cloudbus.cloudsim.DatacenterBroker

- Packages that use DatacenterBroker

  | Package | Description |
  | --- | --- |
  |  |  |
  | --- | --- |
  | org.cloudbus.cloudsim.examples |  |
  | org.cloudbus.cloudsim.examples.power |  |
  | org.cloudbus.cloudsim.power |  |
  | org.workflowsim |  |
- - ### Uses of DatacenterBroker in org.cloudbus.cloudsim.examples

    Methods in org.cloudbus.cloudsim.examples that return DatacenterBroker

    | Modifier and Type | Method and Description |
    | --- | --- |
    |  |  |
    | --- | --- |
    | `DatacenterBroker` | CloudSimExample8.GlobalBroker.`getBroker()` |

    Methods in org.cloudbus.cloudsim.examples with parameters of type DatacenterBroker

    | Modifier and Type | Method and Description |
    | --- | --- |
    |  |  |
    | --- | --- |
    | `protected void` | CloudSimExample8.GlobalBroker.`setBroker(DatacenterBroker broker)` |
  - ### Uses of DatacenterBroker in org.cloudbus.cloudsim.examples.power

    Fields in org.cloudbus.cloudsim.examples.power declared as DatacenterBroker

    | Modifier and Type | Field and Description |
    | --- | --- |
    |  |  |
    | --- | --- |
    | `protected static DatacenterBroker` | RunnerAbstract.`broker` The broker. |

    Methods in org.cloudbus.cloudsim.examples.power that return DatacenterBroker

    | Modifier and Type | Method and Description |
    | --- | --- |
    |  |  |
    | --- | --- |
    | `static DatacenterBroker` | Helper.`createBroker()` Creates the broker. |
  - ### Uses of DatacenterBroker in org.cloudbus.cloudsim.power

    Subclasses of DatacenterBroker in org.cloudbus.cloudsim.power

    | Modifier and Type | Class and Description |
    | --- | --- |
    |  |  |
    | --- | --- |
    | `class` | `PowerDatacenterBroker` A broker for the power package. |
  - ### Uses of DatacenterBroker in org.workflowsim

    Subclasses of DatacenterBroker in org.workflowsim

    | Modifier and Type | Class and Description |
    | --- | --- |
    |  |  |
    | --- | --- |
    | `class` | `WorkflowScheduler` WorkflowScheduler represents a scheduler acting on behalf of a user. |

- Overview
- Package
- Class
- Use
- Tree
- Deprecated
- Index
- Help

- Prev
- Next

- Frames
- No Frames

- All Classes
